# Traditional medicine use in Timor-Leste

**DOI:** 10.1186/s12906-020-02912-9

**Published:** 2020-06-03

**Authors:** Robert Grace, Jacinto Vaz, Julianti Da Costa

**Affiliations:** Department Medicine, Hospital Nacional Guido Valadares, Dili, Timor-Leste

**Keywords:** Traditional medicine, Alternative medicine, Ethnomedicine, Complementary medicine, Timor-Leste

## Abstract

**Background:**

Traditional medicine use is common amongst peoples in developing nations. Timor-Leste is no exception. However little is known about the prevalence, pattern, satisfaction with, cost or type of traditional medicine used in Timor-Leste. This study aims to describe the prevalence, nature and basic demographic factors associated with contemporary traditional medicine use in Timor-Leste.

**Methods:**

A structured interview questionnaire was administered in Tetun to 50 surgical patients, 50 internal medicine patients, 50 hospital staff and 50 hospital visitors at Hospital Nacional Guido Valadares, Timor-Leste’s major referral hospital.

**Results:**

60% of respondents reported having used traditional medicine; 32% within the last year. The greatest use was amongst surgical patients, the least amongst hospital staff. The frequency of traditional medicine use was comparable amongst all groups regardless of size of community, residence or level of education. Traditional medicine use in Timor-Leste is sufficiently common to represent part of the cultural norm. Factors described as promoting traditional medicine use included cost, limited access to other medical services and a belief that traditional medicine was free of side effects. Approximately half the patients reported using traditional medicine for their current illness and many for chronic illness in conjunction with conventional medicine. Conclusion: Traditional medicine use is common in Timor-Leste. Medical practitioners need to be cognizant of the common and potentially concomitant use of traditional medicine alongside conventional medicine when treating patients in Timor-Leste.

## Background

Timor-Leste is a small country in Southeast Asia, still struggling to emerge from the aftermath of a 25-year war of resistance. As a percentage of gross domestic product Timor-Leste is currently thought to spend less on healthcare than any other country [1]. Despite government policy promoting free public healthcare the physical and socio-economic barriers to accessing medical services are substantial. Seventy percent of the population is reported to live in rural or remote areas with limited infrastructure; a quarter of households are more than two hours walk to the nearest health facility [2, 3]. Much of the terrain is mountainous. It was into these mountains that the armed resistance retreated during the Indonesian occupation. As a guerilla outfit unable to leave the forest a working knowledge of traditional medicine and the relevant forest plants was crucial. Collins et al. [4] in their 2007 paper detail many of the plant species employed by the resistance. They highlight the high proportion of tree leaf decoctions, stating that 13 out of 49 administrations were via the oral route as a drink. Infusions, chewing and poultices etc. were all described. Collins et al. [4] further comment that the improving availability of conventional medicine risks the loss of traditional medical knowledge.

Following the withdrawal of Indonesian forces in 1999 state sponsored healthcare virtually collapsed. McWilliam in his article postulates that this allowed a further resurgence of traditional health care [5], in effect filling the gap. Since 1999 modern health services have been slowly improving. However that is not to say traditional medicine has been replaced. Price et al. in their 2016 study found that “most (patients) considered traditional medicine provided an affordable, accessible and acceptable substitute to hospital care” [6]. Little is written about healthcare in Timor-Leste in general, and there is no contemporary data quantifying the rate or pattern of traditional medicine use. Other authors have demonstrated that factors such as education, level of income and place of residence have an impact on the rate of use of traditional medicine [7–9]. This study sought to explore those issues in Timor-Leste; such as whether living in rural versus urban areas affected the rate of use of traditional medicine, or if level of education affected the rate of traditional medicine use and, where possible, the names of those plants used in traditional medicine in Timor-Leste. It further seeks to address the general prevalence, satisfaction, cost and demographic factors associated with traditional medicine use amongst patients, staff and visitors at the Timor-Leste’s main referral hospital, Hospital Nacional Guido Valadares.

## Methods

Following ethics approval and individual verbal consent, a standard structured interview questionnaire was delivered to 200 interviewees. All participants were approached and interviews conducted inside daylight hours at Hospital Nacional Guido Valadares, Dili. Interviewees comprised a convenience sample of four groups. The groups comprised 50 random surgical patients, 50 random internal medicine patients, 50 random hospital staff and 50 random hospital visitors. Persons less than 17, too ill or otherwise unable to consent were excluded. Otherwise all patients, staff and visitors were eligible.

The questionnaire contained both closed and open-ended questions and was delivered in Tetun (the local language) by two Timorese resident medical officers. Close ended questions comprised questions with fixed answers such as gender, age, level of education, where do you live etc. Open-ended questions allowed broader responses such as what types of medicine and plants had they used, cost etc. Traditional medicine was defined in keeping with World Health guidelines as that medicine which comprises the skills and practices based on theories, beliefs and experiences related to indigenous culture of Timor-Leste [10]. All interviews were conducted between September 2018 and January 2019. Interviewees were asked if they had ever used traditional medicine; if so how often, and if they had used it within the last year. They were then asked how much it cost, who prepared it, what form did it take, did they find it effective, why did they take it. Interviewees were then asked where they lived (village, town or city), and their level of education (none, primary, secondary, tertiary). Residence was further examined as being ‘in Dili’ or ‘out of Dili’. (Dili is the capital city of Timor-Leste. It has a population of approximately 250,000, its size dominating Timor-Leste; the entire country has a population of approximately 1.3 million).

Responses to closed-ended questions were analysed quantitatively. Qualitative analysis was applied to open-ended questions. Data was initially recorded by hand and later collated onto an excel spreadsheet. Where relevant data was compared using Pearson’s Chi^2^ test of independence or a two-tailed t-test using Stata v16 statistical software.^1^

## Results

Overall 60% of participants reported having used traditional medicine. Surgical patients had the highest reported rates of usage, 70%, (Table 1). Men were more likely than women to have used traditional medicine. There was no statistical difference in rates of usage of traditional medicine between level of education, size of community or residence in or outside Dili, (Table 1). Of those who had used traditional medicine more than half had used it greater than 10 times, and more than half within the last year, (Table 2).

**Table 1:** Characteristics of Total Sample; Used vs Never Used Traditional Medicine

	Overall		Used Traditional Medicine		Never Used Traditional Medicine		
		n		n		n	*p* value
TOTAL SAMPLE		200		120(60%)		80(40%)	0.04*
Surgical Patients		50		35(70%)		15(30%)	
Internal Med Patients		50		33(66%)		17(34%)	
Hospital Staff		50		22(44%)		28(56%)	
Hospital Visitors		50		30(60%)		20(40%)	
AGE(range)							
Total Sample	41(17–78)	200	42.4(17–77)	120(60%)	38.0(17–78)	80(40%)	0.07**
GENDER							0.01***
Total Male	102			70(69%)		32(31%)	
Total Female	98			50(51%)		48(49%)	
LEVEL OF EDUCATION		200					0.14****
Nil		45		24(53%)		21(47%)	
Primary		25		20(80%)		5(20%)	
Secondary		28		15(54%)		13(46%)	
Tertiary		102		59(58%)		43(42%)	
COMMUNITY SIZE		200					0.53*****
Village		57		32(56%)		25(44%)	
Town		26		18(69%)		8(31%)	
City		117		70(60%)		47(40%)	
PLACE RESIDENCE		200					0.56******
Dili		120		70(58%)		50(42%)	
Outside Dili		80		50(63%)		30(37%)	

*Pearson’s Chi^2^(3) = 8.167; *P* = 0.04

**two tailed t-test t = 1.85; *P* = 0.07

***Pearson’s Chi^2^(1) = 6.456; *P* = 0.01

****Pearson’s Chi^2^(3) = 5.553; *P* = 0.14

*****Pearson’s Chi^2^(2) = 1.278;*P* = 0.53

******Pearson’s Chi^2^(1) = 0.347;*P* = 0.56

**Table 2:** Pattern among users of traditional medicine

	Traditional Medicine Users *n* = 120
Ever Used	120
Only as a child	15(13%)
Used > 10 times	62(52%)
Used in last year	63(53%)
Used for current illness	44(37%)
Used while in hospital	3(3%)

The commonest form of traditional medicine used was a preparation of leaves, fruits or roots consumed as a tea. Leaves and/or bark were occasionally applied to wounds, with the leaves frequently masticated before application. The juice of leaves and fruit were also applied directly to wounds. Types of plants, where known, are contained in Table 3. Massage was also reported.

**Table 3:** Types of Plant Used in Traditional Medicine

LEAVES		BARK		FRUIT	
Unknown	36	Unknown	16	Noni(morinda citfrolia)	3
Guava(psidium guajava)	7	Mankudu	2	Betel Nut(areca catechu)	1
Soursop(annona muricata)	4	Noni	1	Candlenut(alerites molucanna)	1
Mankudu	4			Galangal	1
Noni(morinda citfrolia)	4			Mankudu	1
Tamarind(tamarindus indica)	3	**ROOT**		Marunggi	1
Marunggi	2	Unknown	7	Moriade	1
Ai hanek(Tetun)	1	Ai Dois	1		
Ai sirkada	1	Hudi Bae	1	**OTHER**	
Aitasi(Tetum)	1	Malus Fuik	1	Unknown Oil	2
*Aloe Vera*	1	Mankudu	1	Sarang Semut	2
Avocado(persea americana)	1	Raubi(Makasse)	1	Curcuma Zanthorrhiza	1
Bunga Matahari(Bahasa)Sunflower	1			Garlic	1
Kumis Kucing	1			Ginger	1
Lamtoro(Tetun)	1			Jamu (tree mix from Indonesia)	1
Lemon	1			Jumaris Kuang	1
Malus	1			Kumis Kucing (Flower)	1
Mutahari	1			Oil of coconut(cocos nucifera)	1
Pineapple(ananas comosus)	1				
Sereja	1				
Susai	1				

Forty five percent of those who reported using traditional medicine reported having used it as a pain-relieving agent. Many of the internal medicine patients reported using traditional medicine as part of their treatment for chronic medical conditions such as diabetes, chronic airways disease, kidney and heart failure.

The cost of traditional medicine varied widely. However over 60% of those who used traditional medicine reported it as being free. Cost was otherwise generally less than $10USD but ranged to one reported case of $300USD.

Most traditional medicine was prepared by the patient themselves or family and friends; only 15% of cases reported preparation by a traditional healer.

Commonly reported reasons for using traditional medicine included: it was cheap, the belief it had no side effects, distance from the hospital, word of mouth recommendation from family and friends.

Of those who used traditional medicine 25% reported it did not make them better, 26% reported being unsure, while 49% reported the medicine had made them better.

## Discussion

Traditional medicine use is common in Timor-Leste. A third of survey participants had used traditional medicine within the last year and nearly two thirds had used traditional medicine at some point during their lives. Broadly speaking traditional medicine use is common amongst people of all levels of education, community size and place of residence. This level of traditional medicine use is comparable with other studies from the region and elsewhere [11–13]. Research by Suswardany in neighbouring Indonesia found that respondents used traditional medicine for general health/common illness purposes every day [13]. Survey participants who were patients were, unsurprisingly, more likely to have used traditional medicine than hospital visitors or staff.

Elsewhere others have mooted cost and distance to health facilities as reasons why traditional medicine use might be more frequent in rural communities. Higher levels of education have also been hypothesized as being inversely related to the use of traditional medicine use. However the results presented in Table 1 contrast these findings, as they show no statistical difference in the rates of traditional medicine use between village, town and city dwellers; different levels of education or those who live in or outside of Dili. In short the frequency of use of traditional medicine in Timor-Leste is remarkably consistent across different demographics e.g. place of residence (rural vs city) and level of education. This is surprising. Even those with a tertiary education were found to use traditional medicine at a rate comparable to those who had no formal education.

Why is it that the usual influencers of traditional medicine, i.e. location, education and cost do not seem to be reflected in the patterns of use of traditional medicine in Timor-Leste? Sandberg et al. discuss the adherence by communities to ethnomedicine, placing it the context of a social/cultural practice. Adherence to ethnomedicine forms part of a social network, its use comprising part of the social norm and cultural beliefs of a group [14]. Timor-Leste is a small country with many people living in the city frequently returning to their place of birth in the countryside. Place of origin, family and cultural ties are strong in Timor-Leste. Thus residence within Dili, or secondary town, may not result in as great a diminution of traditional medicine use as might otherwise be expected, i.e. rural versus city dweller take traditional medicine at a similar rate. The observation that recommendation by family or friends is a driver of traditional medicine is also consistent with the concept of traditional medicine being part of a social network. Ideas about disease, illness and treatment are known to be shared through social interaction [12]. Despite Internet access being widely available in Timor-Leste traditional medicine use remains common, possibly as a result of this sociocultural context. Cultural ties, of which traditional medicine use may be considered a part, were especially fostered during the war of resistance, when communities were further isolated and fighters hid in remote areas without access to conventional medicines. Thus the history of traditional medicine use as a sociocultural activity in a group may outweigh other influencers such as location of residence and/or education. Others have commented on the importance of traditional medicines in war-affected areas [15].

In contrast to other studies, [16, 17], where traditional medicine knowledge is held by elders or traditional healers, traditional medicine knowledge in Timor-Leste seems widespread. Most patients, or their family/friends, prepared the medicine themselves. Presumably this keeps costs low, so cost represents little or no deterrent to trying traditional medicine ahead of conventional medicine.

In Timor-Leste the widely held belief that traditional medicine has no side effects is also likely to contribute to its high rate of use. Nearly half of the patient respondents had tried traditional medicine for their current illness. In Anand’s study focusing on epilepsy in Ghana this figure was even higher at 71%. In that study the use of traditional medicine was associated with a significant delay in seeing a recognized medical provider [18]. Interestingly the reported use of traditional medicine by patients when in hospital was very low. It is possible respondents were afraid of upsetting medical staff so under reported their use of traditional medicine while in hospital. This may apply to the study in general, leading to under reporting overall. In keeping with other studies herbal remedies based on leaf, fruit and root extracts were the most popular form of traditional medicine [4]. In the vast majority of instances the plants named in the preparation of the medicine could not be formally identified (Table 3). Naming difficulties resulting from plants having different names in Tetun, Bahasa, varying local dialects, Portuguese and English, further complicated species identification. Only a few of the named plants could be matched with Collins et al’s study [4]. Along with these naming differences Collins et al. also note that different cultural groups in Timor-Leste have significantly different medicinal plant traditions.

The use of traditional medicine as part of a treatment program for chronic disease, such as kidney failure, has been described elsewhere [19, 20]. Traditional medicine has also been described as used in combination with cancer medication in low-middle income countries, [21] and there is nothing to suggest that these practices would not occur in Timor-Leste. Anecdotal observation suggests that traditional bonesetters still practice in Timor-Leste. Collins et al. also refer to this practice [4]. However none of the survey respondents reported using their services. Other research suggests that physical therapies such as bone setting and cutting are among the earliest forms of traditional medicine to give way to conventional medicine [9]. It may be that bone setting as a traditional medical practice is waning.

This study was undertaken at the main referral hospital so does not show the results for Timor-Leste as a whole. This has the potential to create a bias towards city dwellers and more educated people. However Hospital Nacional Guido Valadares is the main referral hospital in what is a highly concentrated health service such that participants in the survey came from all over Timor-Leste. If there were bias towards participants in Dili it might reasonably be argued that, countrywide, traditional medicine may be even more common than demonstrated here.

While no specific data was captured in relation to delays in seeking ‘modern’ medical care, (as a result of first taking traditional medicine), this is a possibility and would be worthy of further investigation. Findings might also be improved by taking participant samples in different locations throughout the country. However this would be a significant undertaking. Information on the income of participants would also have strengthened the study. Finally the fact that resident medical officers undertook the survey may have deterred some participants from fully reporting their traditional medicine use. If the study were to be repeated non-medical personnel delivering the survey might result in even greater reporting of traditional medicine use.

## Conclusion

Traditional medicine use is common in Timor-Leste. Approximately a third of the population has used traditional medicine within the last year, approximately half reporting it to be efficacious. Prior to hospital presentation almost half the patients at Hospital Nacional Guido Valadares have used traditional medicine for their current illness. Many patients use traditional medicine as a treatment for chronic illness, frequently using it in conjunction with conventional medicine. Traditional medicine use in Timor-Leste may form part of shared social and cultural norms promoting belonging to social groups, this may have been particularly important during the war of resistance. Medical practitioners working in Timor-Leste need to be aware of the frequency of traditional medicine use, it’s standing as a social norm in the community and the likelihood that it may be used in combination with conventional medicine.

## Data Availability

The datasets used and/or analysed during the current study are available from the corresponding author on reasonable request.

## Footnotes

1 Stata Statistical Software. Statacorp (Texas USA) 2019. http://www.stata.com
